# Genetic risk factors in melanoma etiopathogenesis and the role of genetic counseling: A concise review

**DOI:** 10.17305/bjbms.2021.7378

**Published:** 2022-04-22

**Authors:** Nikola Šerman, Semir Vranić, Mislav Glibo, Ljiljana Šerman, Zrinka Bukvić Mokos

**Affiliations:** 1 Zagreb Emergency Medicine Service, Zagreb, Croatia; 2 College of Medicine, QU Health, Qatar University, Doha, Qatar; 3 Department of Biology, School of Medicine, University of Zagreb, Zagreb, Croatia; 4 Centre of Excellence in Reproductive and Regenerative Medicine, University of Zagreb School of Medicine, Zagreb, Croatia; 5 School of Medicine, University of Zagreb, Zagreb, Croatia; 6 Department of Dermatology and Venereology, University Hospital Centre Zagreb, Zagreb, Croatia.

**Keywords:** Melanoma, genetics, hereditary syndromes, genetic counseling

## Abstract

Melanoma is a highly aggressive cancer originating from melanocytes. Its etiopathogenesis is strongly related to genetic, epigenetic, and environmental factors. Melanomas encountered in clinical practice are predominantly sporadic, whereas hereditary melanomas account for approximately 10% of the cases. Hereditary melanomas mainly develop due to mutations in the cyclin-dependent kinase 2A (*CDKN2A*) gene, which encodes two tumor suppressor proteins involved in the cell cycle regulation. *CDKN2A*, along with *CDK4*, *TERT*, and *POT1* genes, are high-risk genes for melanoma. Among the genes that carry a moderate risk are *MC1R* and *MITF*, whose protein products are involved in melanin synthesis. The environment also contributes to the development of melanoma. Patients at risk of melanoma should be offered genetic counseling to discuss genetic testing options and the importance of skin UV protection, avoidance of sun exposure, and regular preventive dermatological examinations. Although cancer screening cannot prevent the development of the disease, it allows for early diagnosis when the survival rate is the highest.

## INTRODUCTION

Cutaneous melanoma is a malignant skin tumor that develops from melanocytes that produce melanin. Hippocrates first described melanoma in the 5^th^ century B.C. as a black tumor (*Greek*, *melas* = black, *oma* = tumor); preserved medical texts from the late 16^th^ century also mention incurable black tumors [1].

There are four main histological subtypes of melanomas: Superficial spreading melanoma (70%), nodular melanoma (15-30%), lentigo maligna melanoma (4-10%), and acral lentiginous melanoma (<5%) [2]. In addition to the skin, melanomas may also develop in the eye, upper respiratory, gastrointestinal, and genitourinary systems. Although it accounts for only 5% of all skin cancers, it has the highest mortality rate if not diagnosed early. Its incidence increases annually by 3-7%, and the number of newly diagnosed patients doubles every 10 years, making melanoma the most rapidly increasing cancer diagnosis in the white population [3].

The occurrence of melanoma highly depends on the geographic area, that is, its incidence is the highest in countries with the greatest number of sunny days, such as New Zealand and Australia [4,5]. Therefore, these countries have intensified the primary prevention measures, including education about melanoma and raising awareness about the risk of overexposure to the sun, which has helped reduce the incidence rate [6].

Melanoma risk factors may be classified into three groups: genetic, epigenetic, and environmental [7]. Genetic factors include family history, Fitzpatrick skin Types 1 or 2 (pale skin that easily burns and never tans, and red hair), and defects in DNA repair mechanisms [8]. These risk factors are the main topic of this article, especially genes associated with high or moderate risk of melanoma, hereditary syndromes, and the current genetic counseling approach in at-risk populations.

## GENES THAT INCREASE THE RISK OF MELANOMA

Most malignant tumors in the human body have multifactorial causes, that is, they result from the complex interactions between genes and environment, or in other words, the interplay between genetics and epigenetics [9]. Such tumors are sporadic [10]. They arise from the cells that have accumulated mutations throughout life, eventually leading to their malignant transformation. A small fraction (approximately 10%) of all malignant tumors is hereditary. Unlike sporadic tumors, hereditary tumors occur in persons born with a mutated gene [11]. This phenomenon is called *germline mutation* or malignant variation. It is either inherited from one parent or occurs during gametogenesis, and consequently, a mutated gene is present in every cell of the body [12,13]. However, not everyone who inherits such a mutation will develop melanoma because this also depends on gene penetrance, expressed as a proportion of mutation carriers who develop a disease. For example, if gene penetrance is 100%, all carriers of gene mutation develop the disease; if gene penetrance is 50%, then 50% of mutation carriers develop the disease [14]. Whether or not a gene will have a phenotypical expression depends on other factors that increase or decrease the risk. In the case of melanoma, the other factors include the number of moles and sun exposure [15].

### Cyclin-dependent kinase 2A (*CDKN2A*) gene

It is estimated that ~10% of all melanoma cases diagnosed in 2002 were hereditary, with 40-60% of them occurring due to the mutation of the gene coding for *CDKN2A* [16,17]. William Norris first observed the potential heredity of melanoma in 1820. However, his observation went unnoticed until 1968, when Lynch and Krush first reported on the relationship between pancreatic cancer, multiple moles, and melanoma. Ten years later, Clark described dysplastic nevi in several members of one family and called it the “B-K mole syndrome” [18].

Henry T. Lynch suggested “*familial atypical multiple mole melanoma* (FAMMM)” instead of “B-K mole syndrome.” The first mutation of the *CDKN2A* gene in FAMMM was reported in 1992 [19,20]. FAMMM is inherited as an autosomal dominant trait and is characterized by multiple melanocytic moles (>50 nevi) and positive family history. It is associated with germline mutations of *CDKN2A*. Some mutation carriers may be prone to pancreatic cancer or other malignancies [17].

The *CDKN2A* gene is located at the short arm of chromosome 9 at the 9p21.3 locus. The locus encodes two proteins interacting with two tumor suppressors: Retinoblastoma protein (Rb) and p53 protein (cellular tumor antigen p53 or tumor suppressor p53) [21].

**FIGURE 1:**
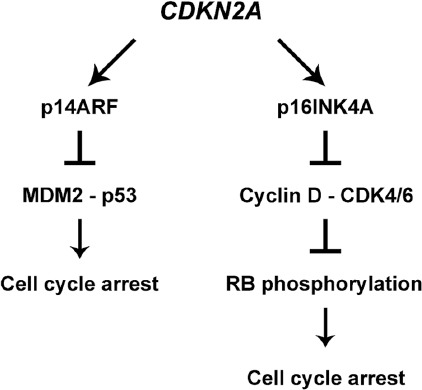
*CDKN2A* gene encodes several different isoforms, of which isoform 4 encodes p14ARF protein while isoform 1 is responsible for p16INK4A protein. Both proteins arrest the cell cycle: p14ARF acts through p53 protein while p16INK4A blocks the Cyclin D/CDK4/6 complex, affecting the pRB phosphorylation.

The gene contains two promoters. When activated, each promoter leads to a different primary transcript, either alpha (α) or beta (β). Each transcript contains a specific exon 1, 1α, and 1β, respectively, whereas they share the exons 2 and 3. The promoter leading to the β transcript is located upstream of the promoter leading to the α transcript. Exon 1 variants being spliced to the shared exon 2 cause the formation of an open reading frame. Thus, exon 2 is read differently due to different starting points in the two transcripts, and the process of translation results in two utterly different proteins. The protein encoded by the α transcript consists of 156 amino acids and is called p16INK4a, while the translation of the β transcript results in the protein called p14ARF, which contains 132 amino acids (Figure 1, Table 1) [22].

**TABLE 1:**
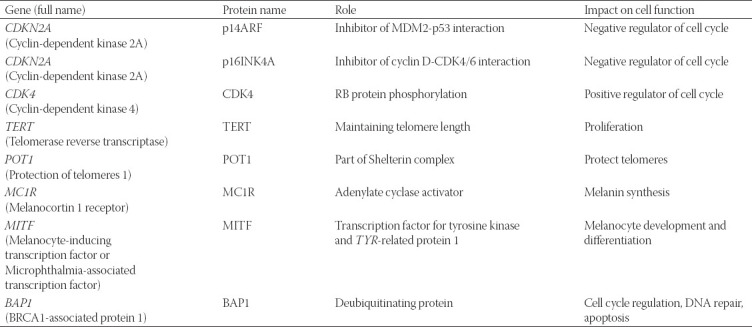
An overview of the genes that are involved in hereditary melanoma susceptibility.

Although different, they both influence the progression of the cell cycle. The most critical control point in the mammalian cell cycle is the G1 phase because it precedes the DNA replication in the S-phase. Thus, the replication of damaged DNA has to be prevented to avoid mutations [23].

The proteins involved in cell cycle regulation belong to two main groups: those that stimulate the cell cycle and those that stop it. The progression of the cell cycle is helped significantly by a group of kinases called CDK, which exert their function by binding to another cyclin protein. After the CDK-cyclin heterodimer is formed, kinase may phosphorylate the target proteins and stimulate the cell cycle [24].

The proteins that stop the cell cycle are called antiproliferative proteins; they are the products of tumor suppressor gene activity. Two well-known tumor suppressor genes are *RB1* and *TP53* [25]. The Rb protein’s function is to halt the cell cycle in the G1 phase, which is accomplished by binding the Rb protein to the E2F transcription factor that stimulates the transcription of many genes responsible for the DNA replication process. In its active state, Rb is unphosphorylated or hypophosphorylated, binds to E2F and stops the cell progression at the restriction point (R-point) in the G1 phase. However, when it is phosphorylated by CDK bound to cyclin, the Rb protein conformation is altered, leading to a release of bound E2F, which triggers the transcription of many genes whose protein products stimulate DNA replication [26].

The p53 protein is also known as “the guardian angel” of the human genome because its expression is increased in cells that suffer DNA damage. It acts as a transcription factor that stimulates *p21* gene transcription [27]. The gene encodes the protein that binds to the CDK-cyclin complex, thus preventing it from phosphorylating the Rb protein and halting the cell cycle. Thus, the damaged DNA should not replicate and create a mutation. At that moment, the cell should wait for repair before it continues the cycle. Moreover, if the repair does not occur, p21 may stimulate the apoptosis of the cell and prevent the occurrence of mutation [28]. p53 in the cell is bound to another protein called mouse double minute 2 homolog (MDM2), which protects it from degradation and becomes active only after being released from the complex.

The protein products of the *CDKN2A* gene exert their activity at the checkpoint of the cell progression from the G1 to S phase. p14ARF inhibits the MDM2 protein and its ubiquitin ligase activity, releasing p53 and making it free to stop the cell cycle through p21 (Figure 1) [29,30].

On the other hand, p16INK4a inhibits the cyclin D-CDK4/6 complex, preventing it from phosphorylating the Rb protein, which then remains active and does not allow E2F protein to transcribe the genes needed for the cell to enter the S-phase, consequently keeping the cell in the G1 phase and not allowing the progression of the cell cycle toward DNA replication (Figure 1) [29,31]. Thus, the two protein products of the *CDKN2A* gene bring the cell cycle to a halt in the same G1 phase by acting through two different mechanisms.

The *CDKN2A* gene mutations have different effects on the synthesis of p16INK4a and p14ARF proteins, as these are formed by transcription resulting from two different reading frames. There are four types of mutations: deletions, insertions, duplications, and substitutions [32]. According to their effects on protein synthesis, they may also be divided into missense, nonsense, and frameshift mutations. The mutation affecting p16INK4a is most frequently located at exon 1a, which corresponds to intron 1, which also harbors ~1/3 of the mutations regarding p14ARF. These mutations can result in an incompletely synthesized protein because the intron mutations may cause the incorrect processing of the primary transcript. Sometimes, the protein is not synthesized because the primary transcript cannot reach the cytoplasm through nuclear pores [33]. Notably, these mutations may increase the efficacy of immune checkpoint inhibitors, such as ipilimumab (anti-CTLA-4 monoclonal antibody) and anti-PD-1 (programmed cell death-1) antibodies pembrolizumab and nivolumab, possibly due to increased mutation load in CDKN2A mutated tumors [34].

### CDK4

*CDK4* is a serine/threonine kinase responsible for the progression of the cell cycle from the G1 to S phase [35]. It exerts its intracellular function only after binding to cyclin D and phosphorylating the retinoblastoma protein at a single point [36]. The result of such monophosphorylation is the release of transcription factor E2F, which triggers the transcription of the cyclin E gene *CCNE1* and its binding to CDK2. The new cyclin E-CDK2 complex additionally hyperphosphorylates the Rb protein at other serine and threonine phosphorylation sites and facilitates the progression of the cell cycle (Table 1) [37,38].

Based on the GenoMEL centers’ study that involved 2137 cutaneous melanoma patients originating from 466 families with at least 3 cutaneous melanoma cases per family, the frequency of the *CDK4* mutations is 2-3% [16].

The *CDK4* gene is located at the short arm of chromosome 12 (12q14). It consists of 8 exons and is mutated in about 4% of melanoma cases [39]. The missense mutation at codon 24 of the second exon triggers the change in the activity of the protein product of this gene from a protooncogene to a dominant oncogene. This change results from histidine (R24H) or cysteine (R24C) being incorporated instead of arginine in codon 24, thus preventing p16 from binding to *CDK4* protein and regulating its activity [40]. The median age of melanoma diagnosis in families with this mutation is 39 years, with an estimated lifetime penetrance of 74% [41].

Sporadic missense and silent mutations of this gene have also been reported in other cancers, such as endometrial cancer. Its expression is altered in ~2% of all cancers, including lung adenocarcinoma, liposarcoma, and glioblastoma. It is also the reason why this mutated form of the *CDK4* gene is a well-chosen target for innovative drugs, such as palbociclib, ribociclib, and abemaciclib (CDK4/6 inhibitors) [42]. Notably, palbociclib has been approved to treat estrogen-positive breast cancer with a high proliferation index (measured by Ki-67), and clinical trials investigating its effectiveness in *CDK4* mutated melanomas are underway [43,44].

### Telomerase reverse transcriptase (*TERT*)

The *TERT* gene encoding the protein part of telomerase reverse transcriptase is located at the short arm of chromosome 5, locus 5p15.33. Telomerase is a ribonucleoprotein that acts as a reverse transcriptase – a function performed by TERT (Table 1). The other ribonucleic part comprises a long non-coding RNA – telomerase RNA (TR or TER) [45,46]. If the cells did not contain telomerase, the chromatids would become ever shorter with every DNA replication because DNA polymerase catalyzes the addition of a new deoxyribonucleoside triphosphate only in a 5’-3’ direction. In other words, it would be beneficial only for one newly synthesized DNA chain. In contrast, the other one would be shorter and shorter with each replication, and this is where telomerase comes into play and prevents such shortening of the chromatids [46]. However, in most cells in the body, telomeres actually do shorten, and the activity of telomerases is needed only in cells such as germ cells, lymphocytes, keratinocytes, endometrial cells, hematopoietic stem cells, and epithelial cells of the intestines, esophagus, and cervix [47]. Maintaining the same length of telomeres is the characteristic of many cancers, including melanoma. Mutations in the *TERT* gene are characteristic of both sporadic and hereditary melanomas. Specific mutations in the promoter of this gene generate the binding site for the family of ETS (*E-twenty-six-specific sequence or E26 transforming sequence*) transformation factors, leading to the increased *TERT* gene transcription [48,49]. The two most common mutations in the gene promoter result from the transition of cytosine to thymine. They are located within 100 bp from the transcription starting site and are called C228T and C250T (chromosome 5, 1,295,228 C>T and 1,295,250 C>T, respectively) [50].

Since these mutations were detected in 77% of intermediate melanocytic tumors and melanoma *in situ* cases, they mark the beginning of malignant transformation [51]. The described mutations in the *TERT* promoter region indicate poorer prognosis and can be used as a marker of shorter survival of these patients [52,53]. Inhibition of the activity of this gene and related protein can be a potential therapeutic target for melanoma patients [54].

### Protection of telomeres protein 1 (*POT1*)

The *POT1* gene is located at the long arm of chromosome 7 (7q31.33). Its protein product, *POT1*, is part of the protective protein complex, *shelterin* or *telosome*, included in the regulation of telomere length, maintenance of chromosomal stability, prevention of aberrant chromosome separation, and protection from unnecessary recombination repair (Table 1) [55]. It is a heterohexamer built of telomeric repeat factor (TRF) 1, TRF2, repressor activator protein 1, TERF1-interacting nuclear factor 2, tripeptidyl-peptidase 1 (TPP1), and POT1 subunits [56]. The POT1 protein consists of 634 amino acids; it is the only part of the complex that can bind directly to a DNA sequence using the oligonucleotide/oligosaccharide-binding (OB) fold domains OB1 and OB2 at the N-terminal. POT1 blocks the function of ataxia telangiectasia and Rad3-related (ART) protein responsible for initiating the DNA break repair through forming a heterodimer with POT1 protein. The heterodimer recruits telomerase to elongate the ends of the chromosome. The same protein may also have the opposite action, that is, prevent the elongation of telomeres by competitive inhibition with telomerase at the 3’ end of a single-stranded DNA molecule. The mutations leading to POT1 inhibition, or mutations resulting in the loss of a binding site for TPP1, increase telomerase activity and are related to various malignancies, including melanoma [57].

According to one study, *POT1* seems to be one of the most commonly mutated genes in hereditary melanoma, along with *CDKN2A* [58]. Moreover, other studies indicated that these mutations were more common than *TERT* mutations [59,60].

### Melanocortin 1 receptor (*MC1R*)

*MC1R* is located at the long arm of chromosome 16 (16q24.3). It encodes the *MC1R*, which belongs to the family of the G protein-coupled receptors (GPCR). The extracellular GPCR domain binds ligands, whereas the intracellular GPCR domain activates adenylyl cyclase and cAMP synthesis through G protein [61]. One of the most critical roles of the *MC1R* is melanin biosynthesis, which occurs in the melanocyte organelles called melanosomes and results from the binding of α-melanocyte-stimulating hormone (αMSH) and agouti signaling protein (Table 1). The binding of αMSH to the MSH receptor (MSH-R) activates adenylyl cyclase, catalyzing cAMP production. The result is the synthesis of eumelanin from tyrosine. ASIP competes for the same receptor, that is, it acts antagonistically by blocking the expression of microphthalmia-associated transcription factor (MITF). MITF is the main factor in melanin synthesis because it regulates the activity of tyrosine-related protein 1 (TRP1) and tyrosinase [62]. The binding of ASIP to MSH-R inhibits eumelanin synthesis and stimulates the production of pheomelanin [63].

Interestingly, *MC1R* variants may substantially increase the penetrance of *CDKN2A* mutations and the risk of melanoma in affected families, particularly multiple MC1R variants and red hair color variants [64]. Identifying polymorphisms and mutations of the *MC1R* gene would enable a better understanding of melanoma susceptibility and potential treatments [65-67].

### Melanocyte-inducing transcription factor or microphthalmia-associated transcription factor (*MITF*)

Melanocyte-inducing transcription factor (*MITF*) gene, or microphthalmia-associated transcription factor gene, is located at the short arm of chromosome 3 (3p13). It encodes the transcription factor called basic-helix-loop-helix-leucine zipper, which it uses to bind to DNA [68]. This domain recognizes specific sequences in the target gene promoters, such as tyrosinase (*TYR*). It is essential for regulating the expression of *TYR* and *TYR*-related proteins, such as *TYR*-related protein 1 and, therefore, plays a central role in regulating melanin synthesis in melanocytes (Table 1) [69]. Among several isoforms of the *MITF* gene, only *MITF-M* is specific for melanocytes [70]. Wnt, TGF-beta, and RTK are only some of the signaling pathways related to the expression of this gene [71].

## MELANOMA-ASSOCIATED SYNDROMES

Melanomas are part of several hereditary syndromes. Two of them, FAMMM and BRCA1-associated protein-1 (*BAP1*) tumor predisposition syndrome, a malignant tumor syndrome (including melanoma) associated with the mutation of the *BAP1* gene, are described in the following paragraphs.

### FAMMM syndrome

The first record of FAMMM syndrome dated from 1820, when Norris described the development of a tumor from a brownish mole that recurred after removal in a patient with about 40 more similar skin lesions and enlarged lymph nodes [72]. The disease was so extensive that the only option was palliative care. The autopsy found that the tumor spread throughout the body, including the heart and lungs. The family history revealed that the patient’s father died of the same disease, and the siblings also had numerous nevi. Norris concluded that it was a hereditary disorder [73].

In 1968, Lynch and Krush described four families with multiple melanomas, including a family where the proband (the first affected family member who seeks medical attention and whose findings raise the suspicion of a hereditary disease) developed the disease at age 26. In 1980, the hereditary nature of this syndrome was confirmed: it showed an autosomal dominant pattern of inheritance. In the 1990s, Lynch reported that the syndrome was associated with other cancers, especially pancreatic carcinoma [74]. The association between this syndrome and pancreatic cancer was explicitly observed in patients carrying the *p16-Leiden* mutation in the *CDKN2A* gene (deletion of 19 base pairs in exon 2 of the gene *CDKN2A*; NM_000077.4: c.225_243del19 (p.p75fs)) [75,76]. The mutation carriers were also prone to esophageal cancer [77,78]. Many other tumors are related to this syndrome, including lung, breast, liver, and brain tumors [79]. The loss of *CDKN2A* heterozygosity is considered the first step in developing melanoma in patients with FAMMM syndrome [80].

Diagnostic criteria for FAMMM syndrome are as follows:

1. Melanoma in one or more first- or second-degree relatives
2. Total body nevi count >50, including atypical nevi (asymmetric, raised above the skin, varying in color, and size)
3. Nevi showing specific histological features, including asymmetry, subepidermal fibroplasia, and lentiginous melanocytic hyperplasia (spindle or epithelioid melanocytes forming nests of different sizes and merging with adjacent rete ridges, and creating bridges), and dermal lymphocyte infiltrates [72].

These patients are referred to genetic counseling, genetic testing, and follow-up. Examination intervals depend on the number of close relatives with the disease and the nevi count. The usual follow-up interval is 6 months. Dermoscopy is the method of choice, but the importance of self-examination should not be underestimated. The use of smartphone applications for examination, which may become available soon, also holds potential [73].

### *BAP1* tumor predisposition syndrome

This syndrome, caused by mutations in the *BAP1* gene, is characterized by uveal melanoma, mesothelioma, and (less often) skin melanoma. Other malignancies may also develop, including kidney, bladder, brain, and soft-tissue tumors [81]. A tumor suppressor gene called *BAP1* codes for ubiquitin carboxy-terminal hydroxylase BAP1. It removes ubiquitin from other proteins, making them more resistant to degradation. It also interferes with their interaction with other proteins. BAP1 is involved in various cellular processes, regulating the cell cycle, transcription, chromatin organization, DNA repair, and apoptosis (Table 1) [82]. This protein, made of 729 amino acids, consists of three main domains: the N-terminal domain that removes ubiquitin, the middle domain that binds the nuclear transcription co-factor called host cell factor 1, and the C-terminal domain that interacts with other proteins [83].

The disease is inherited in an autosomal dominant pattern, and mutations that affect the nuclear localization signal (the sequence of amino acids that directs the protein into the nucleus) or catalytic domain for ubiquitin removal are believed to cause the most severe clinical presentations [84]. *BAP1* gene is often mutated in cases of uveal melanoma, which accounts for 3–5% of all diagnosed melanomas [85]. The carriers of *BAP1* gene mutations are also prone to developing clear cell renal cell carcinoma or mesothelioma [86,87]. More recent studies suggest that *BAP1* mutations may indicate a poorer prognosis for these patients [88].

Mutation of the *BAP1* gene usually manifests as the growth of melanocytic BAP1-associated intradermal tumors (MBAITs). These tumors are raised above the skin surface, are about 5 mm in diameter, and are pigmented or skin-colored. They were previously called atypical Spitz tumors; however, later, it was shown that they differ histologically and morphologically from typical and atypical Spitz tumors. They usually occur in the second decade of life [81]. The number of lesions increases with time but varies from patient to patient [72]. If this gene mutation is suspected, the patient should be referred to genetic counseling, with testing and follow-up measures arranged for the patient and the entire family [89].

## GENETIC COUNSELING

The National Society of Genetic Counselors probably gave the best definition of genetic counseling in 2006: “Genetic counseling is the process of helping people understand and adapt to the medical, psychological, and familial implications of genetic contributions to disease. This process integrates the interpretation to assess the chance of disease, education, and counseling” [90]. The advantage of genetic counseling is to have a better understanding of basic concepts related to genetics, such as mutation, mutation of germinative or somatic cells, early tumor markers, targeted therapy, and molecular analysis [91], and reduction of anxiety related to a possible positive test result and its long-term effect on the patient’s quality of life [92].

The counseling process includes the assessment of disease probability in patients and other members of their families. For that purpose, the consensus on the testing protocol is essential. For example, to test a person for a pathogenic variant of the *CDKN2A* gene, there should be at least three first- or second-degree relatives on the same side of the family with the disease plus a positive prediction test, such as GenoMELPREDICT [93] or evidence of pathogenetic variant of this gene in a family member [94]. When making recommendations, the following factors should be considered: number of family members with a confirmed diagnosis of the skin or ocular melanoma; melanoma before the age of 40; and presence of pancreatic cancer or some other malignancy [95]. These evaluations are best supported by research in families with known mutations such as the mutation *CDKN2A* c.256G > A (Ala86Thr), that is, replacement of guanine by adenine at position 256, resulting in the incorporation of threonine instead of alanine [96].

Genetic counseling is essential not only for the possibility of testing but also for the risk calculation and modification of risk behavior, which play an equally important role in the etiology of the disease since the information received during counseling may positively change the behavior of the person [92]. Specifically, in hereditary melanoma, avoiding prolonged exposure to UV light is of utmost importance [97]. Usually, it is imperative in children not to get tested for adult hereditary tumors. However, in the case of hereditary melanoma, there are indications that genetic testing in children could be justified in the case of *CDKN2A* gene mutations [98]. It was reported that high-quality genetic counseling contributed to a decreased number of hours of UV light exposure in hereditary mutation carriers and non-carriers alike [99].

The person who comes for genetic counseling should be explained the advantages and disadvantages of genetic testing to help them decide whether to accept it. The advantage of early identification of mutation carriers is implementing thorough lifelong monitoring of the carrier and their family members by digital dermoscopy and photography, thus detecting the melanoma in its earliest stage. In the case of *CDKN2A* gene mutation, other malignancies should also be considered, especially pancreatic cancer [17].

The disadvantage of genetic testing is possible anxiety due to the increased risk of melanoma if the test results are positive. It is advisable to refer the mutation carrier to psychological counseling in such cases. A negative test result (absence of mutation), on the other hand, may give a false sense of security, which is also a disadvantage. Therefore, it is essential to highlight that familial malignant melanoma accounts for only 10% of all melanoma cases, whereas the remaining 90% are sporadic. Psychoeducation can help these patients understand the importance of sunscreen use, self-examination, and regular preventive dermatological check-ups.

## CONCLUSIONS

Melanoma is an aggressive malignancy with high metastatic potential. Sporadic melanoma is prevalent in clinical practice, whereas familial malignant melanoma accounts for approximately 10% of the cases. The highest proportion of familial malignant melanoma cases arises from the mutations in the *CDKN2A* gene, which codes for two tumor-suppressor proteins, p14ARF and p16INK4a. Mutations of the *CDKN2A* carry a high risk of melanoma, together with *CDK4*, *TERT*, and *POT1* gene mutations. They encode proteins responsible for cell cycle regulation (*CDKN2A* and *CDK4*) or telomeres length control (*TERT* and *POT1*). The genes that carry a moderate melanoma risk include *MC1R* and *MITF*, whose protein products are involved in melanin synthesis. Since environmental influence plays a role in melanoma development, at-risk patient groups should be offered genetic counseling. During the counseling, along with the option of genetic testing, the patients should be advised to protect their skin from UV light, avoid sun exposure, and keep regular preventive check-ups with their dermatologist. Regular examinations may not prevent the development of the disease, but they increase the probability of early diagnosis when the survival rate is the highest. As none of the above-mentioned genes can be individually held responsible for causing melanoma, the most significant advantage of genetic counseling is psychoeducation.
